# Design of a Device for Sky Light Polarization Measurements

**DOI:** 10.3390/s140814916

**Published:** 2014-08-14

**Authors:** Yujie Wang, Xiaoping Hu, Junxiang Lian, Lilian Zhang, Zhiwen Xian, Tao Ma

**Affiliations:** College of Mechatronics and Automation, National University of Defense Technology, Changsha 410073, Hunan, China; E-Mails: yujiewanghit@gmail.com (Y.W.); jx_Lian@hotmail.com (J.L.); lilian_zhang@hotmail.com (L.Z.); xianzw2011@163.com (Z.X.); tao_ma@live.com (T.M.)

**Keywords:** sky light polarization, least-squares estimation, optimal design, Rayleigh scattering, camera calibration, sun compass

## Abstract

Sky polarization patterns can be used both as indicators of atmospheric turbidity and as a sun compass for navigation. The objective of this study is to improve the precision of sky light polarization measurements by optimal design of the device used. The central part of the system is composed of a Charge Coupled Device (CCD) camera; a fish-eye lens and a linear polarizer. Algorithms for estimating parameters of the polarized light based on three images are derived and the optimal alignments of the polarizer are analyzed. The least-squares estimation is introduced for sky light polarization pattern measurement. The polarization patterns of sky light are obtained using the designed system and they follow almost the same patterns of the single-scattering Rayleigh model. Deviations of polarization angles between observation and the theory are analyzed. The largest deviations occur near the sun and anti-sun directions. Ninety percent of the deviations are less than 5° and 40% percent of them are less than 1°. The deviations decrease evidently as the degree of polarization increases. It also shows that the polarization pattern of the cloudy sky is almost identical as in the blue sky.

## 1. Introduction

Since the discovery of sky light polarization by the French natural philosopher Arago in 1809, it has been studied for many reasons. Sky polarization patterns can be used both as indicators of atmospheric turbidity and as a compass for navigation. The understanding of these optical characteristics has been one of the most interesting and important problems in atmospheric optics [1–4].

The polarization pattern of the sky conveys rich information about the orientation of the sun [5]. There are claims that the Vikings knew of this nearly 1000 years ago and used it for navigation. It is becoming apparent that most animal groups are capable of detecting and using this type of light for a number of different tasks [4,6–8].

When natural sunlight enters the Earth's atmosphere, it is scattered by the air molecules. This scattering is often explained using the Rayleigh scattering theory. The scattered sky light is partially polarized, which means it is a composite of both the natural light and a linearly polarized component [9]. In clear sky, the polarization patterns are quite regular. If the sky is partly clouded, its polarization pattern is rather complex because the polarization of the sky light is disturbed by the clouds [10].

To achieve high navigation accuracy using sky light polarization or to study the atmospheric conditions for weather service purposes, many theoretical and experimental studies have been done on the polarization patterns of sky light [11]. A system based on a Charge Coupled Device (CCD) camera system to measure the natural sky light polarized radiance distribution has been developed in [1]. Lambrinos *et al.* have presented methods of using photosensitive diodes to perceive the polarization pattern and employed a polarized-light compass in a mobile robot [3]. Chu and co-workers enhanced this polarization compass principle [12]. Inspired by the work in [3], another approach using a digital video camera to observe the pattern is presented in [13]. Pust and Shaw have built an imaging Stokes-vector polarimeter using liquid crystal variable retarders to avoid polarization errors in rapidly changing sky conditions [14–16]. Zhang *et al.* have presented a similar imaging polarimeter based on liquid crystal variable retarders [17]. Optimization of polarimeter designs have been addressed in [18–21]. Considering the effect of multiple scattering near the horizon, singularity theory gives a better description of the polarization patterns [22]. The neutral points of the sky light polarization patterns were studied and the fourth neutral polarization point was observed using full-sky imaging polarimetry at higher altitude by air balloon flights in [23,24].

Our purpose here, complementing previous studies on sky light polarization, is to improve the precision of sky light polarization measurements by optimal design of the measuring device and to study the feasibility of navigation based on the polarization patterns of sky light. The rest of this paper is organized as follows: Section 2 presents a description of the system and the optimal alignments of the polarizer. The least-squares estimation is introduced for sky light polarization patterns measurement. Then, the polarization patterns based on the single scattering Rayleigh theory are addressed. In Section 3, the process of the camera calibration is presented and the performance of the system is tested. Next, the polarization patterns of the sky light are obtained using our system and the results are compared with the Rayleigh scattering model. Conclusions are drawn in Section 4.

## 2. Materials and Methods

### 2.1. System Description

**Figure 1.**
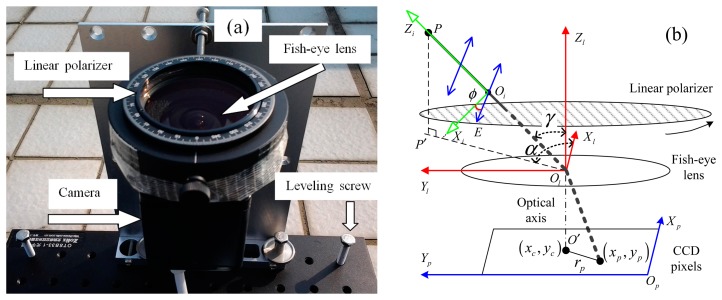
(**a**) The polarization measurement system; (**b**) Schematic representation of the system.

The setup of the polarization measurement system and its schematic representation are shown in Figure 1. The technique used here is similar to the technique described in the several recent publications [1,5,10,24]. The central part of the system is composed of a CCD camera, a fish-eye lens and a linear polarizer. The CCD sensor of the camera is an ICX-445AQA (Sony, Tokyo, Japan), which contains 960 × 1280 active pixels in the image area. The fish-eye lens (*f* = 1.6, focal length 1.8 mm) has an angle of view of 185°. The linear polarizer used in this system is a LPVISE200-A (Thorlabs, Newton, NJ, USA), with highest extinction ratio of up to 9000 at a wavelength of 650 nm. The polarizer is maintained by LM2-A (Thorlabs, Newton, NJ, USA) and LM2-B (Thorlabs, Newton, NJ, USA) optic holders and is rotated manually. With the limitation of the optic holder, which is in front of the fish-eye lens, the angle of view of the camera is restricted to 110°. The right-hand Cartesian coordinate frames shown in Figure 1b used in the article are defined as follows:

*O_p_X_p_Y_p_*: Pixel coordinate frame. Its *X_p_* and *Y_p_* axis is parallel with the row and column of the pixels of the CCD sensor respectively.
*O_l_X_l_Y_l_Z_l_*: Lens coordinate frame. Its *X_l_* and *Y_l_* axis is parallel with *X_p_* and *Y_p_* axis respectively. *Z_l_* axis is in agreement with the optic axis.
*O_i_X_i_Y_i_Z_i_*: Incident rays coordinate frame. Its *Z_i_* axis points at the direction of the incident light and its *X_i_* axis lies in the vertical plane (*O_l_PP*′) containing the direction of the incident light. The axis of *Y_i_* is elided intentionally in Figure 1b for simplicity.

Every pixel (*x_p_*, *y_p_*) of the image corresponds to a particular direction of sky light with the off-axis angle *γ* and the azimuth angle *α*, as is shown in Figure 1b. The relationships of them can be determined by the calibration parameters of the camera (Section 3.1).

During the data collecting process, three or more images are taken with the polarizer on different orientations. These images are combined to acquire the parameters of the incident light which include intensity (*I*), degree of polarization (*d*) and angle of polarization (*ϕ*).

### 2.2. Optimal Alignments of the Polarizer

The polarization pattern of the sky light can be considered of no change in the clear sky with no clouds in a short period of time. The three parameters (*I*, *d*, *ϕ*) of the incident light can be determined by three exposures with the polarizer in different orientations. The algorithms are going to be shown and the optimal alignments of the transmission axis of the polarizer will be sought out in this Section.

For a particular beam of light from the direction of *P*, as is show in Figure 1b, the response of the pixel (*x_p_*, *y_p_*) can be described as:

(1) $$f_{j}(\phi )=\text{KI}\left[1+d\;\cos(2\phi -2\phi_{j})\right],\;j=1,2,3$$

Where *K* is the gain of the CCD sensor. *I* is the intensity of light. *d* is the degree of polarization. *ϕ* is the angle between the polarization direction of incident light and the reference coordinate axis. *ϕ_j_* is the orientation of the transmission axis of polarizer at *j*th exposure.

**Figure 2.**
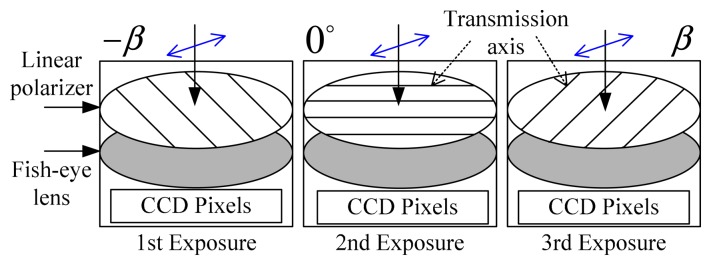
Schematic of the alignments of the polarizer.

For the convenience of analysis, define the orientation of the polarizer axis corresponding to the three exposures is *ϕ*_1_ = −β, *ϕ*_2_ = 0° and *ϕ*_3_ = *β* respectively, as is shown in Figure 2.

Solving Equation (1) we have:

(2) $$\hat{\phi }=\frac{1}{2}\arctan2\left(\frac{f_{3}-f_{1}}{\sin2\beta },\frac{2f_{2}-f_{1}-f_{3}}{1-\cos2\beta }\right)$$

(3) $$\hat{d}=\frac{f_{3}-f_{1}}{(f_{1}+f_{3})\sin2\phi \sin2\beta +(f_{1}-f_{3})\cos2\phi \cos2\beta }$$

What we are going to do is to find the optimal value of *β* that minimizes the estimated error of *ϕ*. Define the criteria function as:

(4) $$\underset{0<\beta <\frac{\pi }{2}}{\min}J=\int_{0}^{\pi }{\sum_{j=1}^{3}\left(\frac{\partial \phi }{\partial f_{j}}\Delta f_{j}\right)}^{2}d\phi$$

where $\frac{\partial \phi }{\partial f_{j}}\Delta f_{j}$ denotes the estimated error of *ϕ* causing by the measurement error Δ*f_j_* at the *j*th exposure. $\int_{0}^{\pi }d\phi$ means summing up the effect of all the incident light with different polarization directions.

Choosing Δ*f*_1_ = Δ*f*_2_ = Δ*f*_3_ = Δ*f*, the criteria function is then given by:

(5) $$\underset{0<\beta <\frac{\pi }{2}}{\min}J=\frac{\pi \Delta f^{2}}{64K^{2}I^{2}d^{2}}\frac{2\sin^{2}\beta -3}{\sin^{4}\beta (\sin^{2}\beta -1)}$$

If *β* → *π*/2, which means the first measurement is correlative with the third measurement, then (sin^2^ *β* −1) → 0, *J* → ∞. If *β* → 0, which means the orientation of the polarizer remains unchanged, then sin^4^ *β* → 0, *J* → ∞.

According to the necessary conditions, we have:

(6) $$\frac{\partial J}{\partial \beta }=\frac{\pi \Delta f^{2}\cos\beta (4\cos^{4}\beta +3\cos^{2}\beta -1)}{-32K^{2}I^{2}d^{2}\sin^{5}\beta {\left(\sin^{2}\beta -1\right)}^{2}}=0$$

Solving Equation (6), the value of *β* is given by:

(7) $$\beta^{*}=\frac{\pi }{3}$$

The sufficient conditions is then given by:

(8) $$\frac{\partial^{2}J}{\partial \beta^{2}}=\frac{\pi \Delta f^{2}(16\sin^{6}\beta -70\sin^{4}\beta +81\sin^{2}\beta -30)}{-32K^{2}I^{2}d^{2}\sin^{6}\beta {\left(\sin^{2}\beta -1\right)}^{2}}\overset{\beta =\frac{\pi }{3}}{=}\frac{20\pi \Delta f^{2}}{9K^{2}I^{2}d^{2}}>0$$

**Figure 3.**
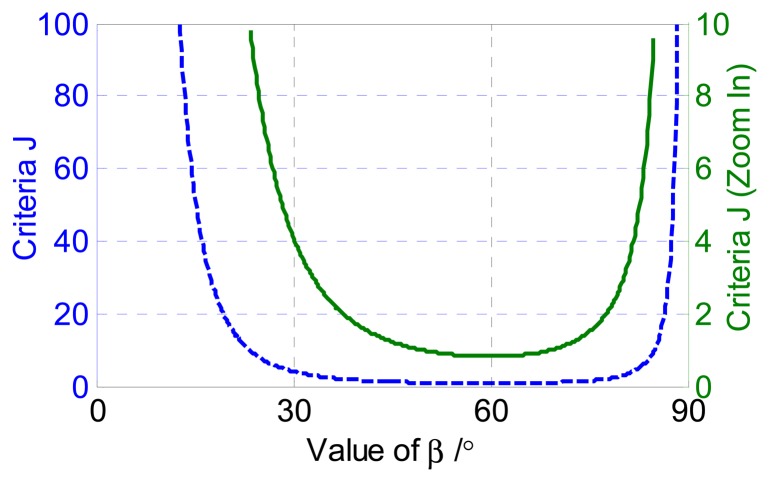
The relationships between the criteria *J* and the value of *β*.

This shows that β* = *π*/3 is the optimal value of *β* that minimizes the estimated error of *ϕ*. This can explain why the visual ﬁelds of the polarization-opponent units (POL-OP units) of some insects were about 60° centered around the zenith [3]. What's more, the period of the polarization direction, which is *π* theoretically, is divided averagely by the three orientations of the polarizer. The results are consistent with previous literatures [18,20]. Assuming Δ*f* = 0.1, *K* = 5/150,000, *I* = 40,000 lux and *d* = 0.6, the relationship between the criteria function *J* and the value of *β* is shown in Figure 3.

Figure 3 shows that the minimum value of criteria *J* corresponds to *β* = *π*/3. If *β* ∈ [38°, 77°], then *J* < 2. As shown in pertinent literatures, *β* was always chosen to be 45° or 60°. The performance of the system at different *β* will be analyzed in detail in Section 3.2.

### 2.3. Least-Squares Estimation of Polarization Patterns

There are some cases in which the orientations of the transmission axis of the polarizer are not inerratic. In addition, more than three exposures could be taken with the linear polarizer in different orientations to minimize the error of parameter estimation. What we are going to do in this Section is to seek the algorithms for parameter estimation using arbitrary number (greater than or equal to three) of measurements with the polarizer in arbitrary different orientations.

Rewrite Equation (1) as follows:

(9) $$f_{j}(\phi )=\text{KId}\cos2\phi \cos2\phi_{j}+\text{KId}\sin2\phi \sin2\phi_{j}+KI$$

For every pixel in CCD sensor, assuming the recorded data contain a sequence of samples *f*_1_, *f*_2_, ⋯, *f_M_* taken with the orientation of the polarizer at *ϕ*_1_, *ϕ*_2_, ⋯, *ϕ_M_*. This can be assign to a kind of problem of fitting sine waves to recorded sine wave data [25].

Define *A*_0_ = *KId* cos 2*ϕ*, *B*_0_ = *KId* sin 2*ϕ* and *C*_0_ = *KI*, then:

(10) $$f_{j}(\phi )=A_{0}\cos2\phi_{j}+B_{0}\sin2\phi_{j}+C_{0}$$

Actually, *A*_0_, *B*_0_ and *C*_0_ are corresponding to the elements of the Stokes vector *s*_0_, *s*_1_ and *s*_2_ respectively [4,14]. In the polarized sky light, the circular polarizations can be neglected [9]. The fourth element of the Stokes vector *s*_3_ is left out in this paper. The degree of polarization (DOP) in this paper is referred as the degree of linear polarization (DOLP) [4,14]. To find the values of *A*_0_, *B*_0_ and *C*_0_, first create the following matrices:

$$D_{0}=\left[\begin{array}{ccc} \cos2\phi_{1} & \sin2\phi_{1} & 1\\ \cos2\phi_{2} & \sin2\phi_{2} & 1\\ \vdots & \vdots & \vdots \\ \cos2\phi_{M} & \sin2\phi_{M} & 1 \end{array}\right],F\left[\begin{array}{c} f_{1}\\ f_{2}\\ \vdots \\ f_{M} \end{array}\right],x_{0}=\left[\begin{array}{c} A_{0}\\ B_{0}\\ C_{0} \end{array}\right]$$

The least-squares estimation of *x̂*_0_ is then given by:

(11) $${\hat{x}}_{0}={\left(D_{0}^{T}D_{0}\right)}^{-1}D_{0}^{T}F$$

To compute the value of *ϕ* and *d*, use:

(12) $$\hat{\phi }=\frac{1}{2}\arctan2[B_{0},A_{0}]$$

(13) $$\hat{d}=\frac{\sqrt{A_{0}^{2}+B_{0}^{2}}}{C_{0}}$$

Assuming *M* = 3, *ϕ*_1_ = −*β*, *ϕ*_2_ = 0 and *ϕ*_3_ = *β*, the estimation of *x̂*_0_ and *ϕ̂* is given by:

(14) $${\hat{x}}_{0}=\left[\begin{array}{c} A_{0}\\ B_{0}\\ C_{0} \end{array}\right]={\left(D_{0}^{T}D_{0}\right)}^{-1}D_{0}^{T}F={\left[\frac{2f_{2}-f_{1}-f_{3}}{2(1-\cos2\beta )}\frac{f_{3}-f_{1}}{2\sin\beta }\frac{f_{1}+f_{3}-2f_{2}\cos2\beta }{2(1-\cos2\beta )}\right]}^{\text{T}}$$

(15) $$\hat{\phi }=\frac{1}{2}\arctan2[B_{0},A_{0}]=\frac{1}{2}\arctan2\left[\frac{f_{3}-f_{1}}{2\sin\beta },\frac{2f_{2}-f_{1}-f_{3}}{2(1-\cos2\beta )}\right]$$

The estimation of *ϕ̂* is the same as Equation (2) in Section 2.2. For one thing, it shows the correctness of two kinds of derived results. For another, the result based on three photos is just a special case of parameter estimation using arbitrary number of measurements with the polarizer in different orientations. To improve the estimation accuracy of sky light polarization patterns, Thirteen pictures were taken with the polarizer on different orientations, *i.e.*, 0°, 30°, 60°, ⋯, 360°, which will be shown in Section 3.3. The algorithms used in the following pages for parameter estimation are based on Equations (12) and (13) in this Section.

### 2.4. Polarization Patterns Based on Rayleigh Scattering Theory

When natural sunlight enters the Earth's atmosphere, it is scattered by small particles. In clear sky, the scattering particles are mainly composed of air molecules and are much smaller than the wavelength of the light striking them. Consequently, the polarization patterns can be explained based on the single-scattering Rayleigh model. Horváth showed that the amount of multiple scattering is strongly affected by atmospheric turbidity and that multiple scattering of light causes depolarization [24]. The wavelength dependency of multiple scattering has also been discussed in [10,24].

**Figure 4.**
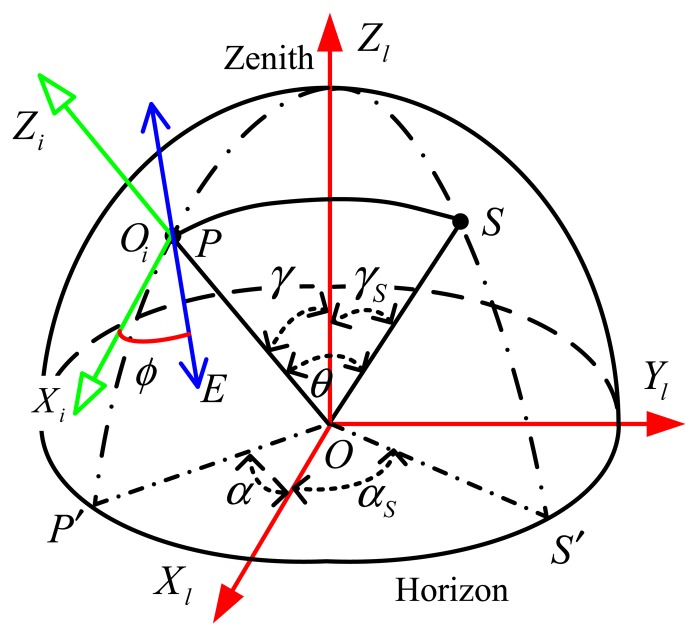
Description of the single scattering Rayleigh model.

The scattered light has an E-vector oriented perpendicular to the plane of the scattering, *i.e.*, perpendicular to the great circle passing through the sun and the point observed, as shown in Figure 4. *O* represents the position of the observer. *S* is the sun position on the celestial sphere with off-axis angle *γ_s_* and the azimuth angle *α_s_*. *P* is the observed direction on the celestial sphere with off-axis angle *γ* and the azimuth angle *α*. *θ* is the scattering angle. *ϕ* is the polarization angle of the scattered light.

The degree of polarization of the scattered light is given by [9]:

(16) $$d=\frac{\sin^{2}\theta }{1+\cos^{2}\theta }$$

The direction of the sun and the direction of the observed point can be expressed in the form of vector coordinate:

(17) $${\vec{OS}}_{l}={\left[\sin\gamma_{s}\cos\alpha_{s}\;\sin\gamma_{s}\sin\alpha_{s}\;\cos\gamma_{s}\right]}^{\text{T}}$$

(18) $${\vec{OP}}_{l}={\left[\sin\gamma \cos\alpha \;\sin\gamma \sin\alpha \;\cos\gamma \right]}^{\text{T}}$$

where the subscript *l* attached to the vector denote the coordinate system in which the vector quantity coordinates are expressed.

The scattering angle *θ* is then given by:

(19) $$\cos\theta ={\vec{OS}}_{l}•{\vec{OP}}_{l}=\sin\gamma_{S}\sin\gamma \cos(\alpha -\alpha_{s})+\cos\gamma_{S}\cos\gamma$$

The polarization angle *ϕ* is the angle between the polarization direction (E-vector) of incident light and the incident rays coordinate axis *X_i_*, as is shown in Figure 4 and Figure 1b.

The polarization direction $\vec{PE}$ can be expressed in frame *l* as follows:

(20) $${\vec{PE}}_{l}={\vec{OS}}_{l}\times {\vec{OP}}_{l}=\left[\begin{array}{c} \sin\alpha_{s}\sin\gamma_{s}\cos\gamma -\sin\alpha \cos\gamma_{s}\sin\gamma \\ \cos\alpha \cos\gamma_{s}\sin\gamma -\cos\alpha_{s}\sin\gamma_{s}\cos\gamma \\ \sin\left(\alpha -\alpha_{s}\right)\sin\gamma_{s}\sin\gamma \end{array}\right]$$

The transformation matrix ${\text{C}}_{l}^{i}$ from coordinate frame *l* to coordinate frame *i* can be carried out as follows [26].

(21) $${\text{C}}_{l}^{i}=\left[\begin{array}{ccc} \cos\gamma & 0 & -\sin\gamma \\ 0 & 1 & 0\\ \sin\gamma & 0 & \cos\gamma \end{array}\right]\left[\begin{array}{ccc} \cos\alpha & \sin\alpha & 0\\ -\sin\alpha & \cos\alpha & 0\\ 0 & 0 & 1 \end{array}\right]$$

The expression of polarization direction $\vec{PE}$ in frame *i* is then given by:

(22) $${\vec{PE}}_{i}={\text{C}}_{l}^{i}{\vec{PE}}_{l}=\left[\begin{array}{c} -\sin(\alpha -\alpha_{s})\sin\gamma_{s}\\ \cos\gamma_{s}\sin\gamma -\sin\gamma_{s}\cos\gamma \cos(\alpha -\alpha_{s})\\ 0 \end{array}\right]$$

The polarization angle *ϕ* is then given by:

(23) $$\tan\phi =\frac{\cos\gamma_{s}\sin\gamma -\sin\gamma_{s}\cos\gamma \cos(\alpha -\alpha_{s})}{-\sin(\alpha -\alpha_{s})\sin\gamma_{s}}$$

Thus, the polarization patterns of the sky light, *i.e.*, the degree of polarization *d* and the polarization angle *ϕ*, based on the single-scattering Rayleigh model, are obtained using Equations (16) and (23).

## 3. Experimental Results and Discussions

### 3.1. Camera Calibration

The internal camera model we used here is very similar to that used in [27,28]. The idea in our approach is to transform the original fish-eye image to follow the pinhole model. The parameters of the distortion model are estimated by forcing straight lines straight after transformation. The camera is calibrated using the Camera Calibration Toolbox for Matlab available online [29]. The internal parameters we defined here is the same as in [29], except the distortion coefficients of the lens. Here, the tangential component of the distortion model is neglected, for that it is less than one percent (about 3‰) of the radial component in our system.

The distortion coefficients *kc* are defined as follows:

(24) $$r_{d}=\left(1+kc\left(1\right)\gamma^{2}+kc\left(2\right)\gamma^{4}+kc\left(3\right)\gamma^{6}+kc\left(4\right)\gamma^{8}\right)\gamma$$

where *γ* is off-axis angle of the incident light. *r_d_* is the normalized projection radius from the principal axis.

**Figure 5.**
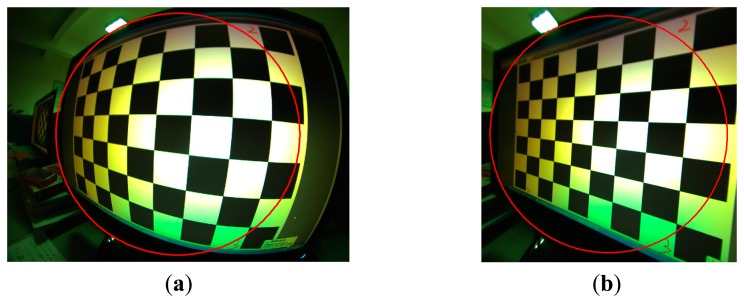
Camera calibration results. (**a**) Original image where the red circle depicts the field of view 110°; (**b**) Undistorted image using the calibration parameters.

The calibration of the camera was performed from ten images of a planar checkerboard pattern which was shown on a screen. The calibration results are illustrated in Figure 5.

The calibration parameters are as follows:

Focal Length: *f_c_* = [479.65 479.73]±[1.60 1.51]
Principal point: [*x_c_* *y_c_*] = [644.95 477.57]±[0.42 0.34]
Skew: *α_c_* = 0.00033 ± 0.00021 ⇒ angle of pixel axes = 89.981° ± 0.012°
Fisheye Distortion: *kc* = [0.02296 −0.02322 0.01347 −0.00347]
Pixel error: *err* = [0.20697 0.20968]

Once the camera is calibrated, the direction of incident light corresponding to the pixel (*x_p_, y_p_*) can be determined. The off-axis angle *γ* and the azimuth angle *α* of the light are given by:

(25) $$\tan\gamma =\frac{\sqrt{{\left(x_{p}-x_{c}\right)}^{2}+{\left(y_{p}-y_{c}\right)}^{2}}}{f_{c}}$$

(26) $$\tan\alpha =\frac{y_{p}-y_{c}}{x_{p}-x_{c}}$$

The nonlinearity of the CCD sensor and the Mueller matrix of the fish-eye lens are ignored at present for the lack of an integrating-sphere uniform-luminance light source. Fortunately, it has been shown that the light polarization patterns were changed slightly after passing through the ﬁsh-eye lens [5].

### 3.2. Performance Test

To evaluate the performance of the system, a liquid crystal display, which is an ideal linear polarization light source, was put in front of the device. The plane of the screen was perpendicular to the optical axis of the device. The polarization patterns of the display were measured by the system. The test was performed at night, with all light turned off to eliminate perturbations. The light source is stable so we have plenty time to take more photos. Twenty four pictures were taken with the polarizer on different orientations, *i.e.*, 0°, 15°, 30°, …, 330°, 345°. When linear polarized lights emit from the liquid crystal display, the polarized directions of them are coincident if observed just along the exit direction. However, the measured polarized directions of them are different, for that almost all the incidences are inclined, and the polarized directions of them will change.

**Figure 6.**
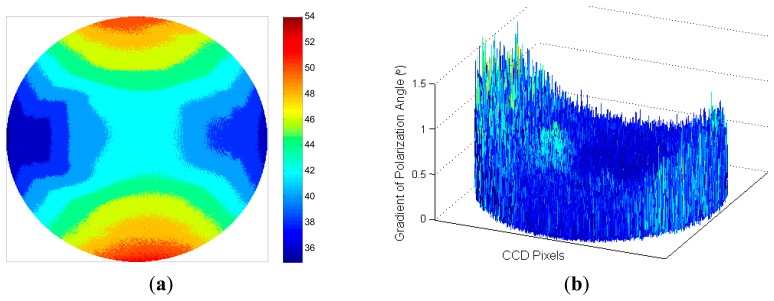
(**a**) Contour plot of measured polarization patterns of the display; (**b**) Gradient of polarization angle at every effective Charge Coupled Device (CCD) pixel.

The polarized direction of every effective incident is computed by Equation (12) using all the 24 pictures, for that the least-squares estimation gives the most accurate polarized directions. The measured polarization patterns of the display are shown in Figure 6a. The polarized directions range from 35° to 55° and are represented by different colors. The polarization patterns show character of axial symmetry and central symmetry. The irregular parts are mainly caused by installation errors and measurement errors.

The absolute values of the gradient of polarization angle at every effective CCD pixels are shown in Figure 6b. The gradient is close to zero in the ideal case because the polarized directions of two bundle of light next to each other should be consistent. The noise of polarization angle, which is defined as the mean value of the gradients at all pixels, is 0.18° in Figure 6b and can be used to evaluate the performance of the system.

At least three photos are necessary to compute the polarization patterns. The optimal alignments of the polarizer have been demonstrated theoretically in Section 2.2. We are going to analyze the performance of the system based on three photos at different *β*, which is the relative angle of polarizer axis at exposure (See Section 2.2). Three pictures with polarizer axis at (*η*_1_, *η*_2_, *η*_3_) were selected each time from the group of 24 pictures according to the following rules, which guarantees each picture has the same weight:

(27) $$\left(\eta_{1},\eta_{2},\eta_{3}\right)=\left(0,\beta ,2\beta \right)+\left(i-1\right)\times 15^{\circ };\;\text{for}\;i=1,2,\cdots ,24;\;\text{for}\;\beta =30^{\circ },45^{\circ },60^{\circ },75^{\circ }$$

**Figure 7.**
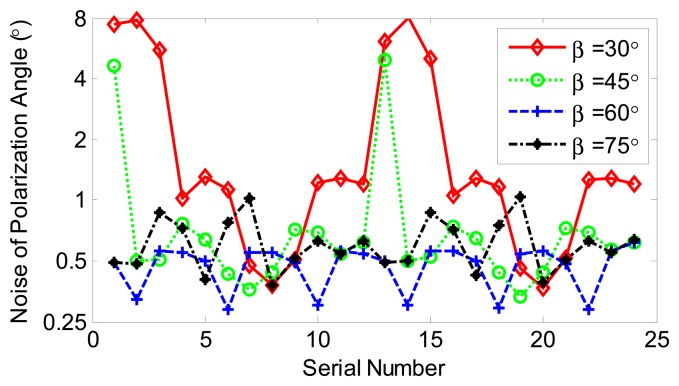
Noise of polarization angle on different *β*.

The noise of polarization angle is computed based on the selected pictures and the results are show in Figure 7. All four curves have a period of 12, which corresponds to the period of polarized light (180°). The noises are obviously larger than the results based on 24 photos. The four curves are all based on three pictures from the group, yet different performances were achieved corresponds to different *β*. The smallest noise was achieved and the results are less insensitive when *β* = 60°, which is consistent with the theoretical analysis in Section 2.2.

Although additional polarizer directions increase the information available for calculating degree of polarization (*d*) and angle of polarization (*ϕ*), it will take much more time for that the polarizer is rotated manually and greater changes of the skylight polarization pattern will occur during the period. A trade-off should be made between the time and the number of measurements. Thirteen pictures were taken in the clear sky, as shown in Section 3.3. The performance of system based on thirteen pictures was tested with the liquid crystal display. It shows that the repeated measuring accuracy of *ϕ* is 0.28°. The error is mainly caused by the noise of the CCD sensor and the orientation error of the polarizer.

### 3.3. Skylight Polarization Measurements

**Figure 8.**
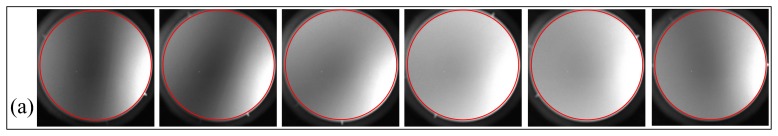

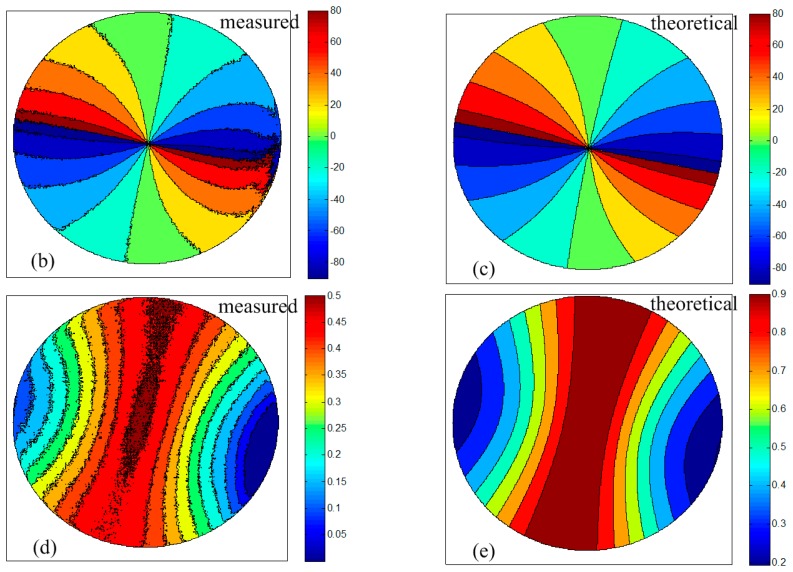
(**a**) Images of the sky with polarizer at 0°, 30°, 60°, 90°, 120°, 150°; (**b**) Contour plot of measured *ϕ̂* of the sky; (**c**) Polarization patterns of *ϕ* based on Rayleigh scattering theory; (**d**) Contour plot of measured *d̂* of the sky; (**e**) Polarization patterns of *d* based on Rayleigh scattering theory.

The polarization measurement system was deployed on the top of a building on the campus of National University of Defense Technology (NUDT) in Changsha on 9 April 2014 at 18:46. Thirteen pictures were taken just before sunset with the polarizer on different orientations, *i.e.*, 0°, 30°, 60°,⋯, 360°. Some of them are shown in Figure 8a. The brightness of the sky obviously changes as the polarizer rotates, which is caused by the polarization of sky light. The measurements were taken in less than one minute (about fifty five seconds), so the polarization patterns of the sky light could be considered to be unchanged within this duration. Only the red part of the spectrum was adopted in the computation in respect that the linear polarizer used in this system is of the highest extinction ratio at wavelength of 650 nm. All images were undistorted using the calibration parameters and were smoothed with a two dimensional Wiener filter to suppress the effect of random noise of the CCD sensor.

The polarization patterns of the skylight measured by our system (*ϕ̂* and *d̂*), are shown in Figure 8b,d. The results are compared with the single-scattering Rayleigh model (*ϕ* and *d*), which are show in Figure 8c,e. The measured polarization patterns *ϕ̂* followed almost the same patterns of the single-scattering Rayleigh model. The measured *d̂* of the sky differs greatly from the theory. The maximum of *d̂* is about 0.5 in this test, which is much less than the theoretical maximum. This is due to the multiple scattering effects. However, the texture characteristics of the degree of polarization are very similar.

The results show that the sun orientation can be evaluated from the polarization patterns of the sky. Thus, the polarization patterns could be used as sun compass without the necessity of seeing the sun. To be precise, the orientation can be achieved with the observation of only a patch of the sky.

**Figure 9.**
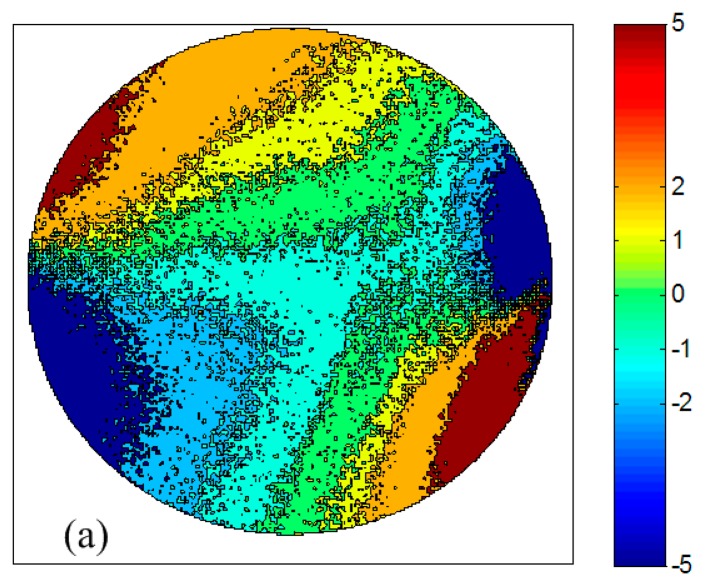

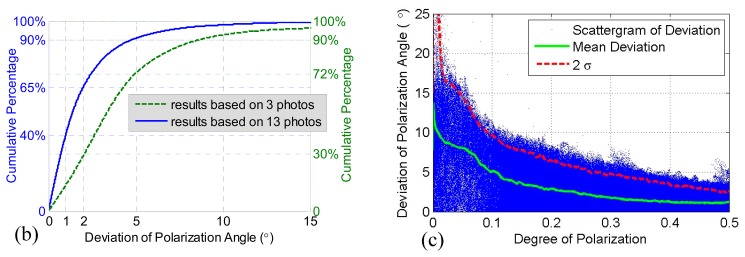
(**a**) Contour plot of the deviations of the polarization angle between observation and theory; (**b**) The statistic characteristics of the deviations; (**c**) The relationships between deviations of the polarization angle and the degree of polarization.

The deviations of the polarization angle *δϕ* between the observation and the theory are shown in Figure 9a and the statistic characteristics of the deviations are shown in Figure 9b by blue solid line. The largest deviations occurred near the directions of the sun and anti-sun, where the degrees of polarization are very low. Ninety percent of the deviations are less than 5° and forty percent of them are less than 1°. The green dashed line in Figure 9b denotes the results based on three photos with the polarizer at 0°, 60°, −60° (*or* 300°), for that the relative angles between them are 60°. The magnitude of the deviations is consistent with previous literature [22]. The comparison of the two curves reflects the superiority of the least-squares estimation based on more photos. The deviations are derived from both measurement error of the polarimetric system and the error of the theoretical model. For one thing, the measurement error may be caused by the nonlinearity and noise of the CCD sensor and the orientation error of the polarizer and so on. For another, the single scattering Rayleigh model may not describe the polarization patterns of the skylight accurately. We will try to find out a better description of the patterns in future work and the multiple scattering theory and experiments in [22,24] enlightened us.

The relationships between deviations of the polarization angle *δϕ* and the degree of polarization *d* are shown in Figure 9c. The deviations decrease evidently as the degrees of polarization increase. The mean deviations are less than 1.6° when the degrees of polarization are greater than 0.3. The degree of polarization (DOP) could be used as a criterion to evaluate the feasibility of navigation based on the polarized skylight.

**Figure 10.**
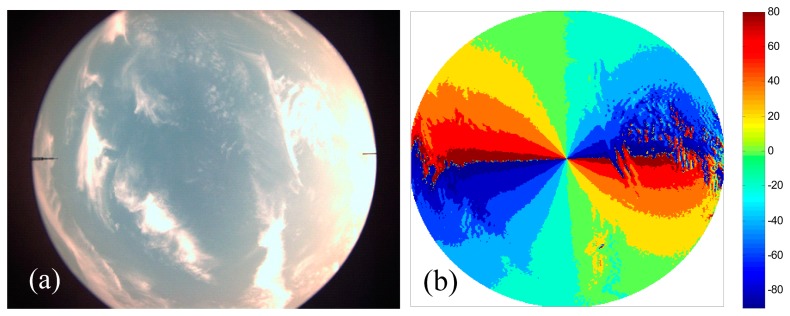
(**a**) Image of the cloudy sky; (**b**) Contour plot of measured *ϕ̂* of the cloudy sky.

Figure 10a shows an image of a partly cloudy sky, which was taken on 11 June 2014 at 19:02 in the same location. The polarization pattern of the cloudy sky is shown in Figure 10b. It shows that the polarization pattern of the cloudy sky is noisier and the deviations are greater, whereas the holistic pattern gives a reliable direction of the sun (or anti-sun) indeed. The polarization pattern of the cloudy sky is almost identical as in the blue sky. The results are consistent with previous literatures [10,15,23]. This shows the feasibility of real time navigation based on polarization pattern of the cloudy sky.

## 4. Conclusions

This paper has presented the design of a device for sky light polarization measurements and the preliminary experiments related to its evaluation. The central part of the system is composed of a CCD camera, a fish-eye lens and a linear polarizer. Algorithms for estimating parameters of the polarized light based on three images are derived and the optimal alignments of the polarizer are proposed. The least-squares estimation has been introduced for sky light polarization patterns measurements using arbitrary number (greater than or equal to three) of photos with the polarizer in arbitrary orientations. The polarization patterns of the sky light are obtained using our system and the results are compared with the single-scattering Rayleigh model. The experimental results show that the measured polarization patterns followed almost the same patterns of the single-scattering Rayleigh model. The largest deviations between observation and the theory occurred near the directions of the sun and anti-sun. 90% of the deviations are less than 5° and 40% of them are less than 1°. The deviations evidently decrease as the degree of polarization increases. The degree of polarization (DOP) could be used as a criterion to evaluate the feasibility of navigation based on the polarized sky light. It also shows that the polarization pattern of the cloudy sky is almost identical as in the blue sky.
